# An ensemble model predicts an upward range shift of the endemic and endangered Yellow‐throated Apalis (*Apalis flavigularis*) under future climate change in Malawi

**DOI:** 10.1002/ece3.11283

**Published:** 2024-04-15

**Authors:** Lumbani Benedicto Banda, Sintayehu W. Dejene, Tiwonge I. Mzumara, Christopher McCarthy, Innocent Pangapanga‐Phiri

**Affiliations:** ^1^ Department of Environment and Natural Resources Management Lilongwe University of Agriculture and Natural Resources (LUANAR) Lilongwe Malawi; ^2^ Africa Centre of Excellence for Climate Smart Agriculture and Biodiversity Conservation Haramaya University Dire Dawa Ethiopia; ^3^ College of Agriculture and Environmental Sciences Haramaya University Dire Dawa Ethiopia; ^4^ Department of Biological Sciences Malawi University of Science and Technology (MUST) Limbe Malawi; ^5^ Zanvyl Krieger School of Arts and Sciences Johns Hopkins University Baltimore Massachusetts USA

**Keywords:** climate change, endangered, endemic, Malawi, species distribution modeling, Yellow‐throated Apalis

## Abstract

Climate change poses a significant threat to endemic and endangered montane bird species with limited elevation and temperature ranges. Understanding their responses to changes in climate is essential for informing conservation actions. This study focused on the montane dwelling Yellow‐throated Apalis (*Apalis flavigularis*) in Malawi, aiming to identify key factors affecting its distribution and predicting its potential distribution under different climate change scenarios. Using an ensemble species distribution modeling approach, we found that the mean temperature of the driest quarter (Bio9), mean temperature of the wettest quarter (Bio8), and precipitation seasonality (Bio15) were the most important variables that influenced the distribution of this species. Across future climate scenarios, the species' geographic range declined where range losses varied from 57.74% (2050 RCP 6.0) to 82.88% (2070 RCP 6.0). We estimate its current range size to be 549 km^2^ which is lower than some previous estimates of its spatial distribution. Moreover, our projections indicate that under future climate scenarios, the species will shift to higher elevations with a large proportion of suitable areas located outside forests, posing challenges for adaptation. Our results suggest that the species may be under greater threat than previously thought; hence, urgent conservation actions are required. We recommend reinforcing the protection of areas predicted to remain suitable under future climate scenarios and the development of a species conservation action plan.

## Graphical abstract

Climate change is expected to have severe effects on endemic and endangered montane species with limited elevation and temperature ranges. Our study focused on the montane dwelling Yellow‐throated Apalis (*Apalis flavigularis*) in Malawi, aiming to predict its potential geographic range under different climate scenarios. Using an ensemble species distribution modeling approach, our study predicted upslope range shift and significant range declines under future climate scenarios, suggesting a higher threat level than previously thought.

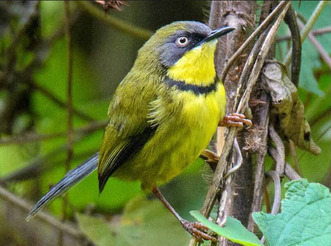

## 1 INTRODUCTION

The effects of climate change on birds are increasingly being documented across the globe (Scridel et al., 2018; Sekercioglu et al., 2012). In a changing climate, some bird species will benefit by experiencing an expansion in their range sizes and increasing their abundance (Davey et al., 2012; Devictor et al., 2008; Kampichler et al., 2012). However, majority of studies indicate that most birds will see a reduction in their range sizes (Brambilla et al., 2018; de Moraes et al., 2020; Hermes et al., 2018; Kiss et al., 2020). Bird species in regions like China (Hu et al., 2020), Europe (Huntley et al., 2008) and south‐central US (Salas et al., 2017) are predicted to shift their geographic ranges in response to changing climate. Within the amazon, predictions suggest that some endemic birds may lose their habitats with some facing the prospect of losing all their habitats under future climate scenarios (de Moraes et al., 2020).

In addition, climate change will cause changes in geographic ranges of bird species at the elevation boundaries. Some species will shift their geographic distributions to higher elevations (upslope) (Auer & King, 2014; Freeman et al., 2018) while others will shift their distribution to lower elevations (downslope) (DeLuca & King, 2017; Foster & D'Amato, 2015). These idiosyncratic responses of species to climate change can be attributed to several factors including the complex interaction among temperature, precipitation (Tingley et al., 2012), species' tolerance to climate (Warren et al., 2001), changes in land use (Srinivasan & Wilcove, 2021), variations in life history traits (Sheldon et al., 2011), and biotic interactions (Araújo et al., 2013).

Tropical montane birds face a heightened vulnerability to climate change because of their narrow geographic ranges (Sekercioglu et al., 2008, 2012). Montane species typically occupy narrow elevation bands (McCain, 2009; Sekercioglu et al., 2012) and are adapted to specific temperature ranges (La Sorte & Jetz, 2010; Sekercioglu et al., 2012), rendering them particularly vulnerable to climate change. As temperatures rise, these species are forced to shift upslope in search for cooler environments. However, this upslope movement results in reduction of available habitat (Colwell et al., 2008; Sekercioglu et al., 2012). Consequently, as species move to higher elevations, their populations often decrease due to reduced habitat size and increased fragmentation (Price et al., 1996; Song et al., 2020). Furthermore, if the climatic niches shift beyond the mountain tops, species may face the threat of extinction (Sekercioglu et al., 2008).

As climate change continues to pose serious challenges for montane bird species, one species deserving particular attention is the Yellow‐throated Apalis (*Apalis flavigularis*). This bird species—endemic and classified as endangered is restricted to the montane habitats of southeastern Malawi. It faces an increasingly uncertain future, primarily due to its confined geographic range, compounded by a range of threats, including climate change and deforestation.

Over the past few decades, the climate in Malawi has undergone noticeable changes, with additional changes projected for the future. Specifically, the temperature in Malawi has increased by approximately 0.9°C, a trend documented between 1960 and 2006 (McSweeney et al., 2010). Projections suggest the possibility of even more pronounced changes, with mean annual temperatures in the country potentially rising by 1.5–5.0°C by the 2090s (McSweeney et al., 2010). Conversely, precipitation patterns for the near future remain uncertain, characterized by substantial variability, including potential declines of up to 13% or remarkable increases of up to 32%. These forecasts also indicate a decrease in dry season rainfall, while the wet season may experience an increase. Adding to these complexities, the projections hint at a rising proportion of total rainfall attributed to intense, heavy rainfall events by the 2060s (McSweeney et al., 2010).

Within this changing climate in Malawi, the Yellow‐throated Apalis becomes greatly threatened especially due to its limited habitat range. Climate change not only alters the suitability of habitats but has the potential to undermine efforts aimed at habitat restoration. Although our understanding of habitat preference for the Yellow‐throated Apalis has improved over time (Mzumara et al., 2012), our knowledge of how the species will be affected by climate change remains low. Nevertheless, projections of future species distributions under changing climate are essential to support and inform conservation efforts.

Species distribution models (SDMs) also known as ecological niche models (ENMs), have proven to be important tools for assessing the impacts of climate change on species distributions (Ahmad et al., 2020; Charitonidou et al., 2022; Kufa et al., 2022). These models predict current and future suitable areas for a given species, aiding in the identification of sites for habitat restoration, and conservation prioritization (Butt et al., 2020; Westwood et al., 2020). By leveraging data on species occurrence and environmental conditions, SDMs characterize a species' environmental tolerance, including its climatic preferences allowing for prediction of potentially suitable areas for a given species (Anderson et al., 2003). Thus, SDMs play a crucial role in identifying areas that meet a species' requirements' while incorporating climate suitability (Bellis et al., 2020).

Evaluating the potential impacts of climate change on species with restricted ranges, especially those classified as endangered is critically important for conservation (Casazza et al., 2014). However, studies investigating the impacts of climate change on these species remain scarce (Dubos et al., 2021; Zhang et al., 2020). The challenges in modeling the present and future distribution of these species are exacerbated by inadequate data for these species which results in small sample sizes and is associated with spatial replication challenges when using climate data (Breiner et al., 2015; Galante et al., 2018). The inadequacies in spatial replicates and small sample sizes often hinder the fulfillment of statistical requirements for species distribution models (SDMs). Nonetheless, our limited knowledge on impacts of climate change on these species hampers our ability to anticipate and prepare for the effects of climate change (Laurance et al., 2011).

In this study, we employ an ensemble of SDMs to predict the extent to which climate change will affect the potential distribution of the endemic and range restricted Yellow‐throated Apalis in Malawi. The study is driven by two objectives: first, to identify the most important environmental variables shaping the distribution of the Yellow‐throated Apalis, and second to predict its potential distribution under different climate scenarios.

## 2 METHODS

### 2.1 Study area

**FIGURE 1:**
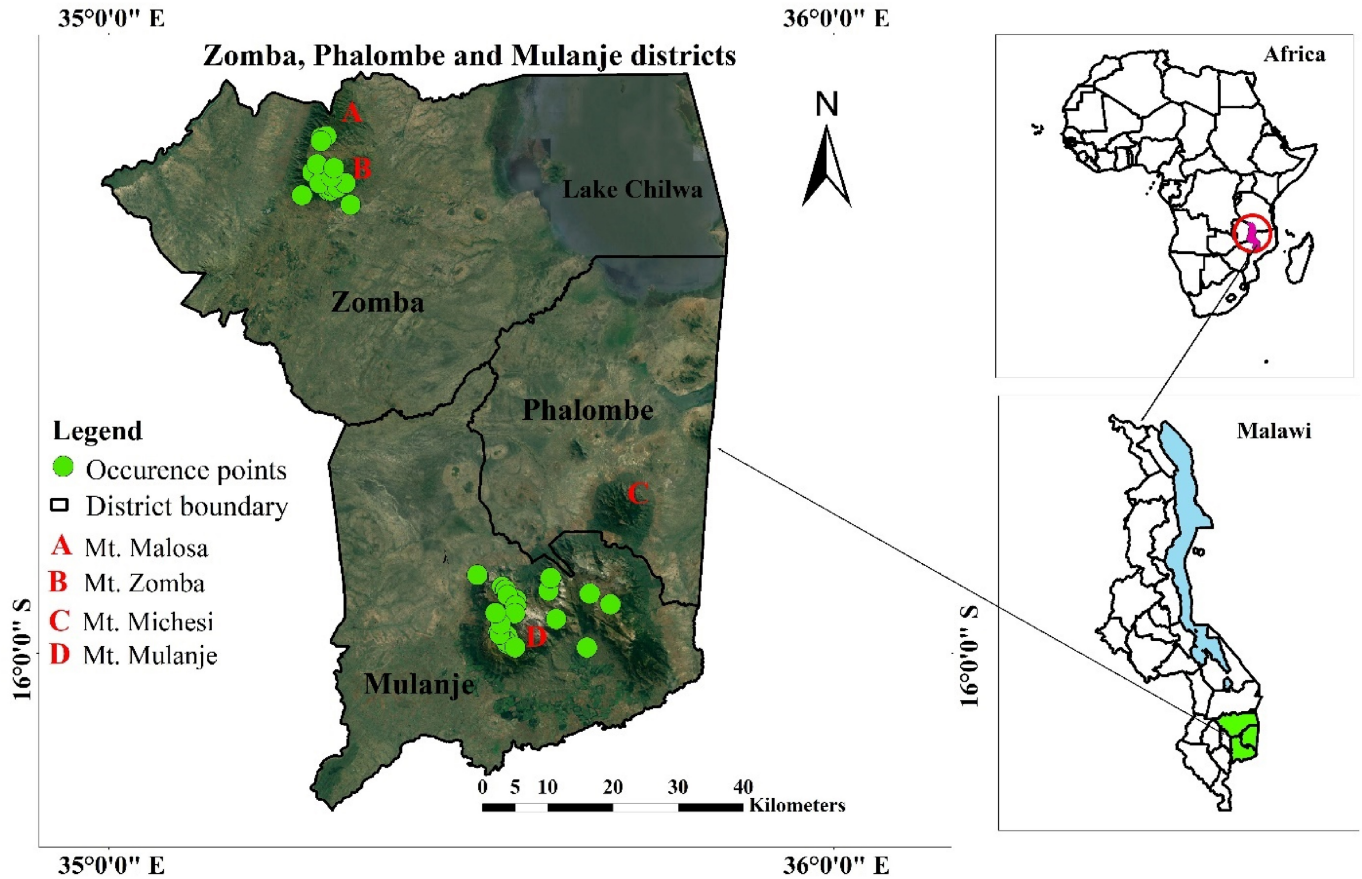
Location of the study area.

The study was conducted in southeastern Malawi, where we targeted mountains in the three districts of Zomba, Mulanje, and Phalombe (Figure 1). Zomba and Mulanje were selected as primary study sites due to the known presence of the Yellow‐throated Apalis in these districts. In Zomba district, the species can be found in the Zomba and Malosa mountains, while in Mulanje district, it inhabits Mulanje Mountain. Additionally, the inclusion of Phalombe district in the study was motivated by historical observations of the species in the Michesi mountain area even though recent surveys have not been conducted there. It is noteworthy that Michesi mountain is geographically close to Mulanje mountain, which suggests the possibility that the species may be dispersing between these neighboring areas.

Within the range of Yellow‐throated Apalis, Mulanje mountain stands out as the largest, covering an area of approximately 650 km^2^ and rising to around 3000 m above sea level (Bayliss et al., 2007). Predominantly, this mountain is a forest reserve with very few areas on the lower slopes under agriculture (Kurzweil, 1992). The landscape of Mulanje mountain is characterized by lowland rainforests extending to 900 m, transitioning to savanna woodlands and Afro‐montane forests between 900 and 1900 m. Grasslands and other Afro‐montane forests appear at high altitudes ranging from 1700 to 2300 m (Kurzweil, 1992). Deforestation and invasive alien species are fundamental problems, with Mexican pine (*Pinus patula*) being the most problematic invasive species (Bayliss et al., 2007). Adjacent to Mulanje Mountain lies the Michesi Mountain in the Phalombe district which extends to about 2105 m above sea level. Michesi mountain is predominantly characterized by miombo woodlands that extend toward Mount Mulanje.

Zomba and Malosa Mountain forest reserves occupy an area of 74 and 110 km^2^, respectively, with elevations reaching 2087 m above sea level. These massifs are separated by the Domasi Valley, which has seen intensive cultivation (Dowsett‐Lemaire & Dowsett, 2006). Montane grasslands and forests are the dominant vegetation types in these massifs (Happold & Happold, 1989). In recent years, natural forests on Zomba mountain have been gradually replaced by pine plantations while the Malosa Forest Reserve has declined due to agricultural encroachment.

The study area experiences climatic conditions typical of Afro‐montane environments. The Mulanje Mountain and Chiperone winds significantly shape rainfall patterns in the district. These two factors result in high rainfall on the windward (South‐East side of Mulanje Mountain) while causing a rain shadow effect on the leeward side (Taulo et al., 2008). Consequently, this results in marked variations in rainfall over relatively short distances (Taulo et al., 2008). The Mulanje district receives an average annual rainfall of 1600 mm, with average annual temperatures ranging from 21 to 23°C, along with maximum temperatures ranging from 32 to 35°C. Phalombe, in contrast experiences a relatively warmer climate, with temperatures varying from 15 to 30°C and annual rainfall averaging 1125 mm. The Zomba District experiences a mean minimum temperature of approximately 11°C and a mean maximum temperature of 26°C and it receives an annual rainfall of 2265 mm (Happold & Happold, 1987).

### 2.2 The study species—Yellow‐throated Apalis

**FIGURE 2:**
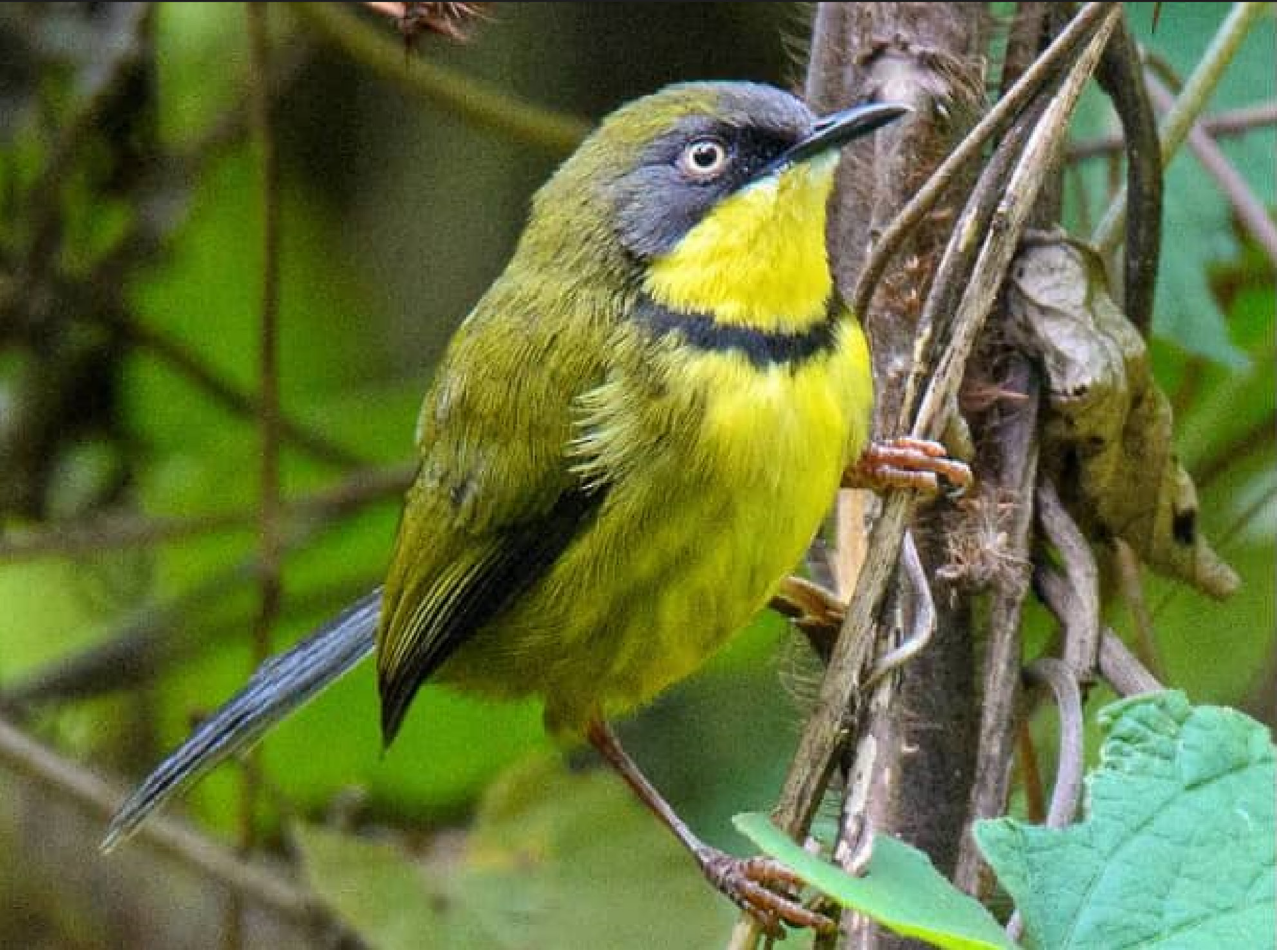
The Yellow‐throated Apalis (*Apalis flavigularis*).

The Yellow‐throated Apalis (*Apalis flavigularis*) is a passerine and the only endemic bird species in Malawi. It has a slender body, with elegant olive‐green underparts. Its most striking feature is the bright yellow throat and breast, bisected by a sleek black bar, creating a captivating contrast (Figure 2). The species is classified as “Endangered” under the IUCN conservation status categories (BirdLife International, 2017). It occurs in southern Malawi where it is restricted to three mountain massifs of Mulanje, Malosa, and Zomba. Yellow‐throated Apalis occurs in evergreen forests at high altitudes (1000–2400 m), though seasonal movements to mid‐altitudes (600–700 m) during the non‐breeding season (Jan–Aug) have been reported (Dowsett‐Lemaire, 1989). This species has an estimated extent of occurrence of approximately 1800 km^2^ (BirdLife International, 2017). Surveys conducted in 2008 estimated the population size of Yellow‐throated Apalis to be at least 7900 individuals on Mulanje mountain alone (Mzumara et al., 2012). Between then and now, its habitats have been subjected to rapid deforestation; hence, population size may be declining (BirdLife International, 2017). The fragmented nature of its habitat and population makes this species particularly vulnerable to anthropogenic activities like deforestation, land use change, and climate change.

### 2.3 Species occurrence data collection and preparation

The study gathered species occurrence records for the Yellow‐throated Apalis from two sources: the Global Biodiversity Information Facility (GBIF) and field surveys. The initial set of occurrence records from GBIF was 39, whereas the initial set from field surveys was 188. GBIF records spanned across different years ranging from 1998 to 2019. Field collection of occurrence data for the Yellow‐throated apalis was conducted from January to March 2019 at the Zomba, Malosa, and Mulanje mountains. The decision to combine data from GBIF and field surveys stemmed from the recognition of the significance of additional occurrence records to improve our analysis. The combined use of historical GBIF data and field survey data allowed us to have adequate data for training our models.

However, not all the records were included in the analysis. Duplicate records were removed from the dataset and occurrence points outside forested areas were excluded. To maintain consistency and avoid overrepresentation of any area we ensured that each 1 km^2^ pixel had only one occurrence record (Polce et al., 2013). After pre‐processing and data cleaning, the final set of occurrence records used for modeling the distribution of Yellow‐throated Apalis consisted of 45 records in total, with 24 records from field surveys and 21 records from GBIF (Table 1).

**TABLE 1:** Occurrence data used in this study.

No	Longitude	Latitude	Source	No	Longitude	Latitude	Source
1	35.54273	−15.907792	Field	25	35.663597	−15.917554	GBIF
2	35.56059	−15.92501	Field	26	35.692177	−15.932337	GBIF
3	35.561829	−15.925907	Field	27	35.659548	−15.991961	GBIF
4	35.54933	−15.98676	Field	28	35.307	−15.3541	GBIF
5	35.55252	−15.983622	Field	29	35.290982	−15.350706	GBIF
6	35.549052	−15.975203	Field	30	35.315782	−15.356426	GBIF
7	35.547381	−15.98295	Field	31	35.3265	−15.3504	GBIF
8	35.537382	−15.971	Field	32	35.6171	−15.9526	GBIF
9	35.539143	−15.973688	Field	33	35.3071	−15.342	GBIF
10	35.30171	−15.35512	Field	34	35.3334	−15.3804	GBIF
11	35.29954	−15.350457	Field	35	35.378461	−15.226958	GBIF
12	35.302549	−15.360546	Field	36	35.287125	−15.324602	GBIF
13	35.281082	−15.334427	Field	37	35.3105	−15.3297	GBIF
14	35.280552	−15.335417	Field	38	35.5605	−15.9917	GBIF
15	35.29318	−15.287476	Field	39	35.370168	−15.217936	GBIF
16	35.300148	−15.285762	Field	40	35.540626	−15.959705	GBIF
17	35.292718	−15.292518	Field	41	35.6065	−15.9135	GBIF
18	35.547267	−15.912433	Field	42	35.6097	−15.8958	GBIF
19	35.54746	−15.91278	Field	43	35.5334	−15.9443	GBIF
20	35.550151	−15.919618	Field	44	35.365933	−15.229845	GBIF
21	35.561437	−15.934198	Field	45	35.508291	−15.891912	GBIF
22	35.560424	−15.94489	Field				
23	35.26647	−15.367278	Field				
24	35.306292	−15.360508	Field				

### 2.4 Environmental variables preparation

The study used 19 bioclimatic variables and one elevation variable at a spatial resolution of 30 arc‐second (~1 km^2^) (Table 2). The study used a fine scale of 1 km^2^ because it was necessary to capture environmental variability that may be lost at coarse resolutions in mountainous areas (Hijmans et al., 2005). In addition, a 1 km^2^ spatial resolution might be appropriate for balancing the goal of deriving patterns at a national scale and informing decisions at local scales (Polce et al., 2013). We downloaded the elevation variable compiled by Fick and Hijmans (2017) from WorldClim database (www.worldclim.org). To generate bioclimatic variables for the current climate scenario we downloaded monthly precipitation, minimum temperature, and maximum temperature data for the years 1998 through 2016 from Chelsa (Karger & Zimmermann, 2018). From these data, we computed monthly averages for the entire period and used these averages to calculate bioclimatic variables. We computed 19 bioclimatic variables using the "biovars" function of the "dismo" package (Hijmans et al., 2017) in R open‐source software (R Core Team, 2019). Thus, our current climate data represents averages from 1998 to 2016.

**TABLE 2:** Environmental variables and their corresponding codes.

Code	Bioclimatic variable	Code	Bioclimatic variable
Bio1	Annual mean temperature	Bio11	Mean Temperature of Coldest Quarter
Bio2	Mean diurnal range	Bio12	Annual Precipitation
Bio3	Isothermality	Bio13	Precipitation of Wettest Month
Bio4	Temperature seasonality	Bio14	Precipitation of Driest Month
Bio5	Maximum temperature of the warmest month	Bio15	Precipitation Seasonality
Bio6	Minimum temperature of coldest month	Bio16	Precipitation of Wettest Quarter
Bio7	Temperature Annual Range	Bio17	Precipitation of Driest Quarter
Bio 8	Mean Temperature of Wettest Quarter	Bio18	Precipitation of Warmest Quarter
Bio 9	Mean Temperature of Driest Quarter	Bio19	Precipitation of Coldest Quarter
Bio10	Mean Temperature of Warmest Quarter	Elevation	Elevation

General circulation models (GCMs) were used to select variables for future climate scenarios. GCMs have many uncertainties, which result in variations in their projections. Variations among GCMs projections arise because of differences in model formulation, the response of the modeled climate and its spatial structure, and the internal variability of climate systems (Van den Hurk et al., 2014). Hence, the choice of the GCM is one of the primary sources of variability in projections resulting from species distribution models (Thuiller et al., 2019). To account for these variations, we averaged three GCMs (MIROC‐ESM‐CHEM, MIROC5, and MIROC‐ESM) and made projections based on the averages. To select these GCM models, we compared ten GCMs from the Coupled Model Intercomparison Project 5 (CMIP5) using GCM compareR (Fajardo et al., 2020). GCM compareR compares GCMs using either a reference baseline climate or an average of the projections from all selected GCMs (ensemble) contrasting layers (Fajardo et al., 2020). This study used the latter and selected GCMs based on the average distances from the ensemble, where models with the least distances were preferable.

The selected GCMs were used to extract representative concentration pathways (RCPs) ‐ RCP 4.5, RCP 6.0, and RCP 8.5, for the years 2050 and 2070. RCPs represent a range of climate outcomes that result from a combination of factors, such as greenhouse gases, aerosol concentrations, and land use change (Van Vuuren et al., 2011). The RCPs indicate the expected amount of radiative forcing that will occur by the end of the 21st century. Radiative forcing is measured in Watts per square meter (W/m^2^), and represents the extra heat retained by the lower atmosphere due to increased levels of greenhouse gases. There are four RCP scenarios – 2.6, 4.5, 6.0 and 8.5 where each corresponds to differential increases in radiative forcing values compared to industrial levels. The RCP scenarios are projected to result in rising temperatures, with RCP 2.6 leading to an increase of 0.3–1.7°C, RCP 4.5 leading to 1.1–2.6°C, RCP 6.0 leading to 1.4–3.1°C, and RCP 8.5 leading to 2.6–4.8°C. (Collins et al., 2013). In this study, we did not include RCP 2.6, because given the current policy projections, the feasibility of RCP 2.6 is doubtful (De Sousa et al., 2017).

Our initial selection comprised 20 environmental variables, consisting of 19 derived bioclimatic variables and one elevation variable (Table 2). We processed these variables to minimize multicollinearity and selected a final set of environmental variables for modeling. When fitting a model, it is important to ensure that the explanatory variables are not correlated. Multicollinearity can make it difficult to interpret the relationship between independent and response variables (Heikkinen et al., 2006; Zurell et al., 2020) and increases the uncertainty in species distribution models (Junior & Nobrega, 2018). To reduce multicollinearity among environmental variables, we adopted a procedure recommended by Guisan et al. (2017). Initially, we conducted a principal component analysis (PCA) among the environmental variables in R software using the "ade4" package (Dray & Dufour, 2007). This allowed us to identify and retain those variables that explained the highest variation within the species occurrence point space. After conducting PCA, we used the "usdm" package (Naimi et al., 2014) to calculate the variance inflation factor (VIF). Variables with a VIF < 10 were selected for inclusion in the analysis (Erfanian et al., 2021).

### 2.5 Species distribution modeling

Some SDMs require presence and absence data; however, in this study, we only used presence or occurrence data. Therefore, we randomly generated pseudo‐absence data, which are also known as the background samples. Some model algorithms are sensitive to the background sample selection. When background samples are selected from large distances from known occurrences, the model may have good discriminative ability for regional conditions and poor ability to discriminate fine‐scale conditions that limit species distribution (VanDerWal et al., 2009). Because of the fragmented habitats for Yellow‐throated Apalis, we avoided the selection of background points from the entire study area. Background points were selected within a certain geographical distance from the presence points (VanDerWal et al., 2009). Background points were sampled randomly within a 15 km radius of the presence points. The choice of sampling radius was based on our knowledge of the areas that could have suitable abiotic conditions for Yellow‐throated Apalis and where the species could potentially disperse. When predicting the fundamental niche, pseudo‐absences need to be drawn from areas that have suitable abiotic conditions but are within the dispersal possibilities of the target species (Soberon & Peterson, 2005). For all the model algorithms, we generated 1000 background samples.

When modeling species distribution, an important consideration that needs to be made is the choice of the modeling algorithm. The predictions of species distribution models can be uncertain under different model algorithms because the algorithm used influences the outcome (Dai et al., 2017; Wright et al., 2015). Nevertheless, ensemble models that combine different individual model algorithms have the potential to reduce bias in the prediction of species distribution models (Araújo & New, 2007). In this study, we adopted an ensemble modeling technique using the "biomod2" package (Thuiller et al., 2014). Ten modeling algorithms were used in this study: artificial neural network (ANN), classification tree analysis (CTA), flexible discriminant analysis (FDA), generalized additive models (GAM), generalized boosting modeling (GBM), generalized linear modeling (GLM), maximum entropy (MAXENT, Phillips), multivariate adaptive regression splines (MARS), Random Forest (RF), and surface range envelope (SRE). For each modeling algorithm, the occurrence data were randomly split into two groups: 70% for training and 30% for testing the model. The ideal situation would have been to use data from GBIF for training the model and data from field surveys for model testing. However, because we had few occurrence records, we preferred to train the model with more data by combining data from the GBIF and field observations.

For each SDM algorithm, we had ten evaluation runs and three sets of pseudo‐absences (background samples), resulting in a total of 300 SDMs (10 algorithms × 10 evaluation runs × 3 sets of background samples). We evaluated the model performance using the True Skill Statistic (TSS) and the Area Under the Curve (AUC) of the receiver operating curves (ROCs). For ensemble modeling, only models with a mean TSS ≥ 0.7 among all evaluations were selected. The final binary maps of the potential distribution of the species for each period were determined using TSS. We used the cut‐off value determined by "biomod2" as the threshold for transforming the SDM outputs into presence and absence. Values higher than the threshold represented “presence or suitable areas” whereas values lower than the threshold represented “absence or unsuitable areas”.

#### 2.5.1 Spatial habitat changes in potential distribution

Predictions of potential habitats for Yellow‐throated Apalis from the ensemble model under each future climate scenario were overlaid on top of the predictions for current climate scenarios. Areas suitable for both current and future scenarios were categorized as “stable areas.” Areas that were suitable under the current climate but not suitable under future climate scenarios were classified as “lost areas” (Thuiller et al., 2014). All areas that were classified as unsuitable under the current climate, but suitable under future climate scenarios, were classified as gained areas. We further computed the size of suitable areas under each climate scenarios using the “BIOMOD_RangeSize” function from the "biomod2" package.

The study also examined the extent to which the areas projected to be suitable for the Yellow‐throated Apalis were situated within forested areas. This was necessary due to the species' reliance on forest habitats. This was conducted by overlaying the current forested areas onto the areas projected to be suitable for the species under different climate scenarios. We classified a Landsat 8 image from 2019 (Acquisition date: 20/07/2019; Path: 167; Row: 071) into three main categories: water bodies, forests, and other land cover types. Using this classification, we isolated the forested regions and overlaid them on the projected suitability areas for the Yellow‐throated Apalis to determine how much of the suitable habitat overlapped with forested regions. Given that the Yellow‐throated Apalis primarily inhabits forests, our analysis specifically concentrated on these forested areas. Additionally, we calculated the size of the suitable areas within forests to ascertain the proportion of the projected suitable areas crucial for the species' conservation efforts. Even though the current forested areas may not be realistic under future climate scenarios they may still provide valuable insights regarding the extent to which climatically suitable areas correspond to habitat requirements.

#### 2.5.2 Altitudinal shifts in potential distribution

To test the altitudinal shift of the distribution of the Yellow‐throated Apalis under future climate scenarios, we used elevation data from WorldClim. The probability maps of the potential distribution of Yellow‐throated Apalis were converted into presence/absence maps using a cut‐off value generated by the "bimod2" package in R. Using these maps, we extracted elevation values from the altitude raster for areas that corresponded to presence points for each climate scenario. We used these data to calculate the mean altitude at presence points for the species under different climate scenarios. To examine if there were statistically significant shifts in altitude for current and future climate scenarios, we performed the Kruskal–Wallis test followed by post‐hoc Dunn's test.

The study also investigated whether the centroids of the potential distribution of the Yellow‐throated Apalis under future climate scenarios will change by analyzing changes in the centroids of suitable areas. We calculated these centroids using ArcGIS 10.5. Due to the fragmented nature of the Yellow‐throated Apalis' distribution, computing a single centroid for the entire region might yield less meaningful results, particularly when attempting to associate them with specific suitable regions for the species. To address this, for each climate scenario we calculated separate centroids for the Zomba‐Malosa and Mulanje mountain massifs. This approach ensured that the centroids were region‐specific and accurately represented the geometric center of the potential distribution of the species for each mountain massif. We further computed distances between the centroid of current suitability and suitability values for all future climate scenarios.

## 3 RESULTS

### 3.1 Model performance

All models had good predictive performance except for the SRE, which had the worst (Table 3). The GBM, FDA, MAXENT, and GLM respectively were among the best performing algorithms. The TSS values for individual algorithms varied from 0.798 (GBM) to 0.535 (SRE) and the AUC values ranged from 0.920 (GBM) to 0.767 (SRE). We selected eight algorithms for ensemble projection: GBM, FDA, MAXENT, GLM, RF, ANN, MARS, and CTA. Our selection targeted algorithms that had a mean TSS value greater or equal to 0.7; hence, we excluded the GAM and SRE algorithms. The ensemble model performed better than all individual models, with a TSS value of 0.858 and an AUC value of 0.974.

**TABLE 3:** Evaluation statistics for each modeling algorithm and the ensemble model.

Model	TSS	ROC
Generalized boosting model (GBM)	0.798	0.920
Flexible discriminant analysis (FDA)	0.790	0.895
Maximum entropy (MAXENT)	0.784	0.890
Generalized linear model (GLM)	0.772	0.914
Random Forest (RF)	0.769	0.913
Artificial neural network (ANN)	0.752	0.891
Multiple adaptive regression splines (MARS)	0.751	0.877
Classification tree analysis (CTA)	0.705	0.848
Generalized additive model (GAM)	0.687	0.847
Surface range envelope (SRE)	0.535	0.767
Ensemble model	0.858	0.974

### 3.2 Variable contribution

Following a procedure described in section 2.4, we selected four environmental variables for inclusion into our analysis. These variables included mean temperature of driest quarter (Bio9), mean temperature of wettest quarter (Bio8), precipitation seasonality (Bio15) and annual precipitation (Bio12). Our models included these four independent variables to predict the potential distribution of Yellow‐throated Apalis. Among these, the most important variables in predicting the potential distribution of Yellow‐throated Apalis were mean temperature of the driest quarter (Bio9), mean temperature of wettest quarter (Bio8) and precipitation seasonality (Bio15) (Table 4). However, outcomes of our analysis point an interesting pattern: temperature emerged as more influential than precipitation for predicting the distribution of Yellow‐throated Apalis. The mean temperature of driest quarter (Bio9) had the highest influence, contributing 46.93% to the prediction. In contrast, the combined contribution of annual precipitation (Bio12; 13.94%) and precipitation seasonality (Bio15; 17.58%) was relatively less (31.45%). This finding emphasizes the significance of temperature‐related variables in defining habitat range for the Yellow‐throated Apalis.

**TABLE 4:** Mean variable importance of four variables included in the ensemble model for predicting potential distribution of the Yellow‐throated Apalis.

Variable	Mean variable importance	Percentage
Mean temperature of driest quarter (bio9)	0.678	46.93
Mean temperature of wettest quarter (bio8)	0.311	21.55
Precipitation seasonality (bio15)	0.254	17.58
Annual precipitation (bio12)	0.201	13.94

### 3.3 Current and future potential distributions

**FIGURE 3:**
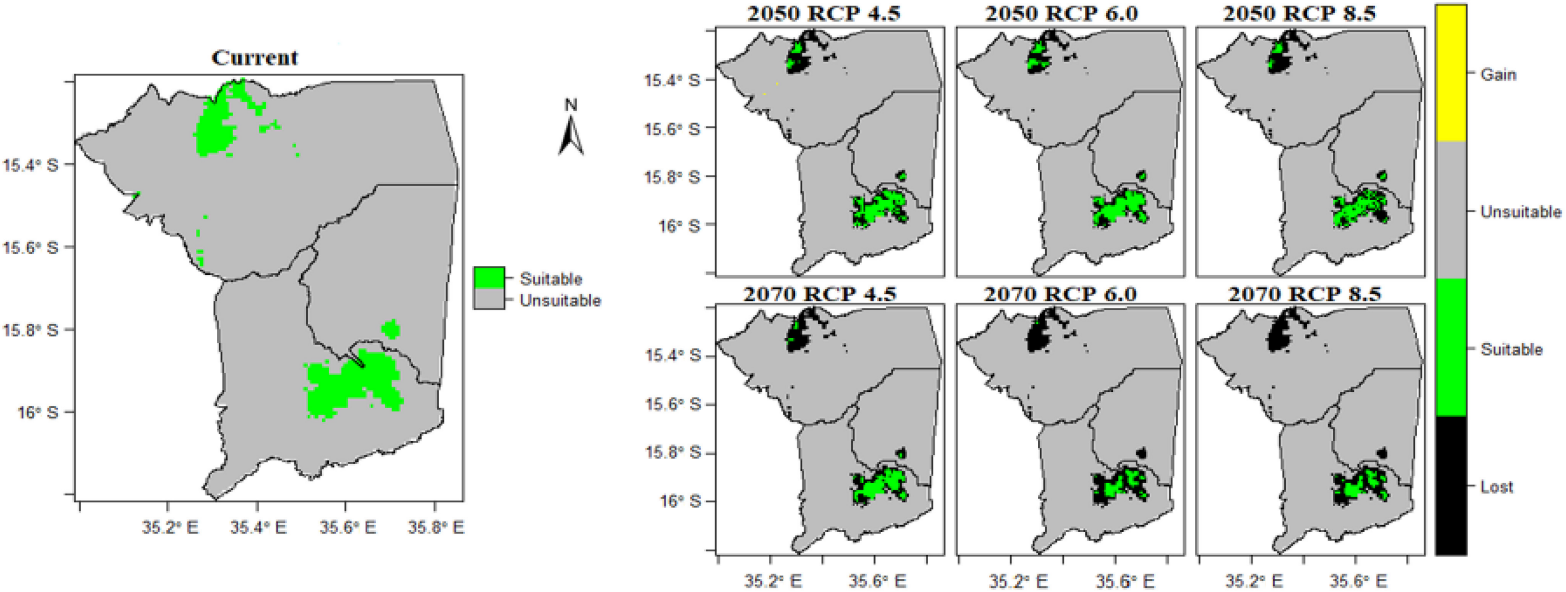
Projected potential distribution of the Yellow‐throated Apalis (*Apalis flavigularis*) under current and six future climate scenarios: 2050 RCP 4.5, 2050 RCP 6.0, 2050 RCP 8.5, 2070 RCP 4.5, 2070 RCP 6.0 and 2070 RCP 8.5.

The ensemble model's predictions of the current distribution of the Yellow‐throated Apalis were consistent with our knowledge of the species' distribution. The model predicted suitable climates for this species at Mulanje, Zomba, Malosa and Michesi mountains (Figure 3). Nevertheless, our predictions suggest that the distribution of the Yellow‐throated Apalis will undergo a significant decline under future climate scenarios (Figure 3; Table 5). The results suggest that the Yellow‐throated Apalis will experience huge geographic range losses varying from 57.74% to 82.88%. Under current climate conditions, the species had a suitable area of about 549 km^2^. However, our predictions indicate that this suitable area will decrease, with the worst declines being experienced under RCP 6.0 scenario in 2070, where only 94 km^2^ is projected to remain. The results further reveal that under future climate scenarios Yellow‐throated Apalis will generally not gain novel suitable areas. Notably, for all future scenarios no gains in suitable areas were projected except for the RCP 4.5 scenario of 2050. Unfortunately, these gains in suitable area were very small (3 km^2^) and will not compensate for the losses. Despite the high projected range losses for the species, the projected available geographic ranges under future climate scenarios were projected to be generally stable.

**TABLE 5:** The predicted potential suitable areas for the Yellow‐throated Apalis (*Apalis flavigularis*) under current and future climate scenarios.

	Current	2050			2070		
		RCP 4.5	RCP 6.0	RCP 8.5	RCP 4.5	RCP 6.0	RCP 8.5
Range size (km^2^)	549	211	232	184	180	94	101
Gain (km^2^)		3	0	0	0	0	0
%loss		62.11	57.74	66.49	67.21	82.88	81.60
%Stable		208	232	184	180	94	101
%Gain		0.55	0	0	0	0	0
Species net range change (%)		−61.57	−57.74	−66.49	−67.21	−82.88	−81.60

*Note*: Stable represents the area that is currently suitable for the species and predicted to remain suitable in future. Gain represents areas not currently suitable for the species but predicted to be suitable in future.

Additionally, our findings reveal that among the different mountain massifs, suitable areas for the Yellow‐throated Apalis will be least affected on Mulanje mountain than in all other mountains. Mulanje mountain is the only location which retains suitable areas for the species under all climate scenarios considered in this study. On the other hand, Zomba, Malosa and Michesi mountains lose most of the suitable areas for the species under future climate scenarios. These areas lose all of the suitable areas or retain very small suitable areas for the Yellow‐throated Apalis under all climate scenarios in 2070.

#### 3.3.1 Forested area coverage within projected suitability

**FIGURE 4:**
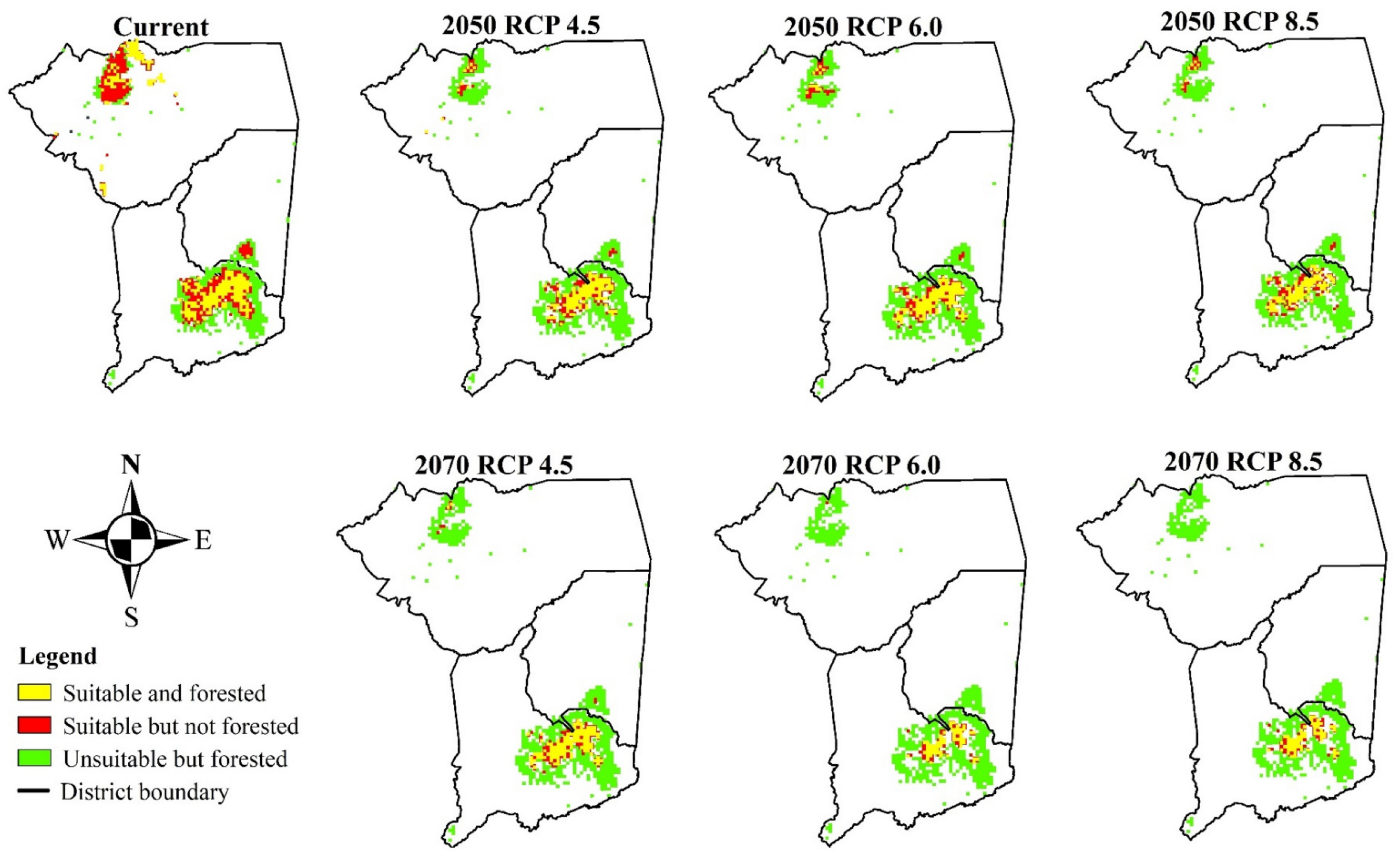
Projected suitable areas for Yellow‐throated Apalis situated within current forested regions.

The projected suitable areas for the Yellow‐throated Apalis within forested regions were smaller across both the current and all future climate scenarios (Figure 4). While all projected suitable areas for the Yellow‐throated Apalis were situated within protected areas, most of these suitable areas were outside the forested regions under current and future climate scenarios. Of the total 549 km^2^ projected to be suitable for the species under current climate scenarios, only 229.99 km^2^ were situated within forested areas representing approximately 42% of the total area projected to be suitable (Table 6). Similarly, for all future climate scenarios the size of projected suitable areas located within forests was substantially smaller, ranging from 54.49 km^2^ (2070 RCP 6.0) to 116.54 km^2^ (2050 RCP 6.0). Overall, under most climate scenarios, more than half of the areas projected to be suitable for the species were located outside forested areas.

**TABLE 6:** Size of projected suitable area for Yellow‐throated Apalis under forested and unforested areas.

	Current	2050			2070		
		RCP4.5	RCP6.0	RCP8.5	RCP4.5	RCP6.0	RCP8.5
Total projected suitable (km^2^)	549	211	232	184	180	94	101
Suitable and Forested (km^2^)	229.99	105.51	116.54	92.43	106.95	54.49	57.67
Suitable and Forested (%)	41.89	50.00	50.23	50.23	59.42	57.97	57.1
Suitable but unforested (km^2^)	319.01	105.49	115.46	91.57	73.05	39.51	43.33
Suitable but unforested (%)	58.11	50.00	49.77	49.77	40.58	42.03	42.9

#### 3.3.2 Elevational shifts in potential distribution

The results showed that suitable areas for the Yellow‐throated Apalis will shift to high elevation under future climate scenarios. The Kruskal–Wallis' test showed significant differences (Chi square = 441.39, *p* = <.001, df = 6) in the median altitude of predicted suitable areas among different climate scenarios. The post‐hoc analysis using Dunn test showed that the altitude or elevation for suitable areas under current climate was statistically significantly lower than all future climate scenarios (*p* < .05). Under the current climate, suitable areas for the species had a mean altitude of 1751.508 m a.s.l (Table 7). While under future climate scenarios, the species suitable areas had mean altitude ranging from 2124.93 (2050 RCP8.5) to 2349.61 m a.s.l (2070 RCP 6.0).

**TABLE 7:** Mean and median elevation for the projected current and future potential distribution of the Yellow‐throated Apalis.

Period	Climate scenario	Mean elevation (m)	Median elevation (m)
Present	Current	1751.508	1825.0^a^
2050	RCP 4.5	2127.640	2109.0^b^
	RCP 6.0	2134.491	2121.0^b^
	RCP 8.5	2124.929	2096.0^b^
2070	RCP 4.5	2211.022	2197.5^c^
	RCP 6.0	2349.606	2321.0^d^
	RCP 8.5	2327.703	2306.0^d^

*Note*: Median elevation values that do not share a superscript letter are significantly different (p < 0.05) while those that share superscript letters are not significantly different (p > 0.05).

**FIGURE 5:**
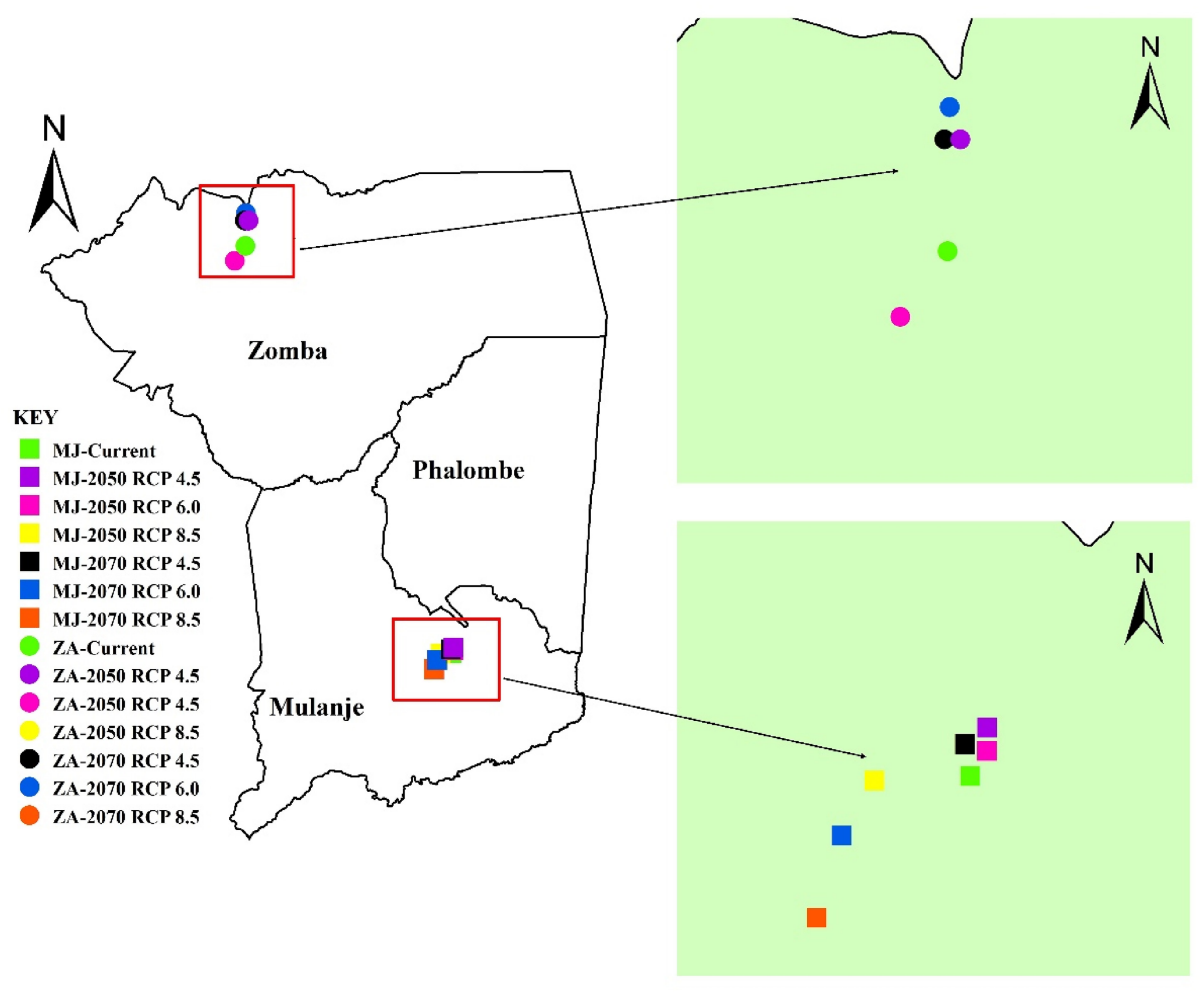
Centroids for current and future projected distributions of Yellow‐throated Apalis. Points labeled “MJ” correspond to Mulanje mountain, while points labeled “ZM” correspond to Zomba‐Malosa mountains.

The assessment of centroid shifts in suitability for the Yellow‐throated Apalis under various climate scenarios revealed minor changes in centroids within the Mulanje and Zomba‐Malosa mountain regions. In both the Mulanje, and the Zomba‐Malosa mountain regions, the centroids exhibit minor shifts, primarily toward the north (Figure 5). An examination of the distances between the current suitability centroid and future suitability centroids indicates that these centroid shifts remain moderate. In most instances, the centroids remain within 5 km of the current suitability centroid, except for the RCP 6.0 scenario of 2070 on Zomba‐Malosa mountain, where more than 5 km shifts were projected (Table 8). A comparison of these shifts between Mulanje and Zomba‐Malosa regions suggests that higher centroid shifts are expected on the Zomba‐Malosa regions than on Mulanje. On Mulanje region the centroid shifts varied from 0.68 to 3.78 km while on Zomba‐Malosa region these shifts ranged from 3.05 to 5.58 km.

**TABLE 8:** Centroid shifts of suitability for Yellow‐throated Apalis under different climate scenarios.

Climate scenario	Mulanje Mountain		Zomba‐Malosa Mountains	
	Distance from current suitability centroid (km)	Direction of shift	Distance from current suitability centroid (km)	Direction of shift
2050 RCP 4.5	1.15	North	4.3	North
2050 RCP 6.0	0.69	North	3.05	South‐West
2050 RCP 8.5	1.82	West	4.33	North
2070 RCP 4.5	0.68	North	4.28	North
2070 RCP 6.0	2.46	West‐South	5.58	North
2070 RCP 8.5	3.78	South‐West	NA	NA

## 4 DISCUSSION

### 4.1 Range reductions for the Yellow‐throated Apalis under changing climate

Our study highlights the potential vulnerability of the Yellow‐throated Apalis to climate change. The findings suggest that the Yellow‐throated Apalis will experience severe geographic range reductions under future climate change scenarios. These projected range reductions are consistent with predictions made for other tropical montane bird species in various parts of the world (Avalos & Hernández, 2015; Velásquez‐Tibatá et al., 2012; Vieira de Souza et al., 2011). Our findings further suggest that temperature is an important factor in determining the geographic range of the Yellow‐throated Apalis. Consequently, the future range losses predicted for this species may be associated with future changes in temperature. This is because the most important variable affecting the distribution of this species was the mean temperature of driest quarter (Bio9).

Temperature dynamics play a crucial role in dictating range shifts, particularly for high elevation species like the Yellow‐throated Apalis. Such species are inclined to shift their habitats in response to changing temperature conditions, whereas those dwelling in lower elevations tend to shift in response to changing precipitation conditions (Tingley et al., 2009). The mechanisms through which temperature affects the geographic ranges of birds are diverse, encompassing disruptions in nesting behavior and reduced reproductive success (Sherry et al., 2015; Townsend et al., 2013) and changes in the availability of food resources (Flousek et al., 2015; Hermes et al., 2018).

Furthermore, our study predicts an upslope shift in the potential distribution of Yellow‐throated Apalis under future climate scenarios. This result aligns with findings from other regions, such as the central European mountains (Flousek et al., 2015) and Sierra Nevada Mountains (Tingley et al., 2012), where birds have responded to rising temperatures by moving to higher altitudes. Increasing temperatures can cause species to shift their range to higher altitudes in the search for colder climates, inevitably shrinking their geographic distributions due to spatial limitations at higher elevations (Buermann et al., 2011; La Sorte & Jetz, 2010), and potentially leading to populations decline (Harris et al., 2014). As the elevation range of a bird species narrows, the risk of extinction increases (Tingley et al., 2012). Consequently, the upward range shift driven by temperature increase may render some species, especially those endemic to tropical highlands, vulnerable to local extinction (La Sorte & Jetz, 2010). Tropical birds are particularly vulnerable because of their limited thermal adaptability, stemming from lower climate variability when compared to species in temperate regions (Ghalambor et al., 2006). This reduced capacity to adapt to climate change complicates their prospects for survival. The extent to which the Yellow‐throated Apalis can adapt to climate change remains uncertain, and the species may face additional challenges in adaptation due to the fragmented nature of its habitats.

In addition, the study predicts a significantly lower current range size of the Yellow‐throated Apalis (549 km^2^) than previously estimated (1800 km^2^) by the IUCN (Bird Life International, 2017). Estimates by IUCN are generally optimistic because they are based on extent of occurrence (EOO) and do not take into account other important variables like climate that influence species distribution. Conversely, alternative range size estimations by Tobias et al. (2022) suggest a range size of 513.16 km^2^ for the Yellow‐throated Apalis which is comparable to estimates from our study. These estimates though slightly lower than those observed in this study are less optimistic as they are not based on extent of occurrence. The similarity between these estimates and those from this study highlights the robustness of our predictions. The small range size of the Yellow‐throated Apalis highlights the localized nature of the species' distribution emphasizing the need for targeted conservation efforts. Given the limited geographic range projected in this study, immediate conservation actions are required. This is critically important, considering that globally threatened bird species with small geographic ranges (<20,000 km^2^) are under great threat from climate change, even when full dispersal scenarios are considered (Avalos & Hernández, 2015).

Apart from having a small geographic range size and declines in geographic range under future climate scenarios, the study has revealed that most of the climatically suitable regions for the Yellow‐throated Apalis are outside forested areas. Considering that this species is a forest species, these findings are worrying as they highlight the mismatch between Yellow‐throated Apalis's climate preferences and availability of its preferred habitat. Of the total projected suitable climate of 549 km^2^ under current climate only approximately only 230 km^2^ was within forested areas. The situation was even worse for future climate scenarios where the species is projected to have very small range sizes. Within these small geographic range sizes, at least 50% of the projected suitable regions were outside forested areas under all future climate scenarios. This result emphasizes the vulnerability of the species to changing climate conditions. One noteworthy element was the size of suitable area that remained within forested regions under future climate scenarios within each mountain region. Of the two mountain regions Mulanje mountain retained higher proportions of suitable areas within forests than the Zomba and Malosa regions under current and future climate scenarios. This implies that the Mulanje mountain is the most important conservation area for ensuring the persistence of the populations of this species.

The analysis of centroid shifts in suitability for the Yellow‐throated Apalis under various climate scenarios provides valuable insights into the species' potential response to changing environmental conditions within the Mulanje and Zomba‐Malosa mountain regions. The results suggest that there will be minimal shift in the centroids for suitable areas under various future climate scenarios where most of the centroids are projected to remain within 5 km of the current suitability centroid. This coupled with failure to gain new suitable areas under future climate scenarios highlights the critical threat of extinction that the species faces and the need for protecting existing habitats.

Even though our findings suggest that Yellow‐throated Apalis faces a heightened risk of extinction due to habitat loss driven by climate change, it is essential to acknowledge that shifts in species distribution are influenced by a complex web of factors beyond just climate. These interconnected elements include changes in land use, habitat fragmentation, and various biotic factors. Research on other taxa has shown that variations in topography, leading to microclimate heterogeneity, can lessen the risk of species extirpation by providing microrefugia (Suggitt et al., 2018). The presence of a range of microclimates acts as a protective barrier against the detrimental impacts of climate change, offering refuge areas where species are more likely to endure (Somero, 2010; Suggitt et al., 2018). While these findings highlight the crucial role of microclimate heterogeneity in species survival amid changing climates, it is important to recognize that our study did not include other vital factors, such as changes in land use and habitat fragmentation. Consequently, the actual extinction risk to Yellow‐throated Apalis may be influenced by a combination of these unexplored variables.

In addition, other factors that we did not include in our models, such as deforestation and competition, may restrict the range size of the Yellow‐throated Apalis. This implies that the actual distribution of this species may be lower than predicted in this study. This is especially important considering that forest habitats in which this species occurs have declined by at least 50% between 1995 and 2015 and will continue to decrease in the future (Banda, 2020). Therefore, even though suitable climates for this species may be available under future climate scenarios, it is not clear whether relevant vegetation habitats will be available. In this study we did not include other factors such as land use and vegetation health, because these factors are likely to change with climate. Thus, currently available maps of these factors are not realistic under future climate scenarios. Furthermore, other factors such as population, dispersal, and physiological and evolutionary characteristics can also affect species under climate change conditions (Foden et al., 2013; Thuiller et al., 2005). However, climate change is a major threat to endemic birds that occur at higher altitudes than deforestation (Harris et al., 2014), because at high altitudes, human influence is relatively lower than at mid and lower altitudes.

### 4.2 Conservation implications

This study suggests that climate change will affect the endangered and endemic Yellow‐throated Apalis by reducing its geographic range size while shifting its climatic niche upslope. Based on the high range reductions predicted in this study (57.74%–82.88%), the species requires urgent conservation actions. The impacts of climate change extend beyond the immediate threat to the existence of the Yellow‐throated Apalis; they also interfere with the intricate dynamics of its trophic niche. As elucidated by Tobias et al. (2022), the Yellow‐throated Apalis is classified as an invertivore, a category that highlights its crucial role in the local ecosystem. The Yellow‐throated Apalis, by virtue of its trophic niche, may play a crucial role in pest control within the region. Its predation on insects contributes to the ecological balance, limiting potential outbreaks of pest species that could threaten local vegetation and agricultural crops. The loss of this species could thus compromise the ecosystem services it provides.

The effects of climate change may act in combination with other threats such as habitat destruction through deforestation. Conservation of the Yellow‐throated Apalis will therefore require integrated approaches combining habitat protection with climate change adaptation and mitigation strategies. The protection of areas that are predicted to remain suitable under future climate change scenarios will be crucial for ensuring the persistence of species populations.

Implementing various conservation actions for the Yellow‐throated Apalis is difficult at present because it does not have a species conservation action plan. Although this species occurs in protected areas, general conservation actions may not be adequate. Therefore, this study recommends the development of a species conservation action plan for the Yellow‐throated Apalis. This plan should focus not only on enhancing the protection of important habitats for the species but also establishing long‐term population monitoring initiatives that will indicate the effectiveness of various conservation activities. Implementation of these recommendations will require collaboration among different stakeholders that have other ongoing activities in the habitats of the Yellow‐throated Apalis.

## 5 CONCLUSION

In this study, we successfully predicted the potential impact of climate change on the distribution of the Yellow‐throated Apalis. Our results reveal that variables such as the mean temperature of the driest quarter, mean temperature of the wettest quarter, and precipitation seasonality play significant roles in influencing the distribution of the species. Notably, the mean temperature of the driest quarter emerges as the most influential variable. Furthermore, our research highlights the negative impacts of climate change on the Yellow‐throated Apalis, as it is expected to experience a decline in its geographic range under future climate change scenarios. These findings provide valuable insights into identifying regions that are likely to remain suitable for the species in future climate change scenarios. To ensure the survival of the Yellow‐throated Apalis, it is crucial to enhance the protection of its habitats. We recommend the development and implementation of a species conservation action plan, which will serve as a guiding framework for conservation efforts aimed at protecting this species.

## AUTHOR CONTRIBUTIONS

**Lumbani Benedicto Banda:** Conceptualization (lead); data curation (lead); formal analysis (lead); funding acquisition (lead); investigation (lead); methodology (lead); project administration (lead); resources (equal); software (lead); supervision (supporting); validation (lead); visualization (lead); writing – original draft (lead); writing – review and editing (lead). **Sintayehu W. Dejene:** Conceptualization (equal); funding acquisition (equal); investigation (equal); methodology (equal); project administration (equal); resources (equal); supervision (lead); validation (equal); writing – review and editing (equal). **Tiwonge I. Mzumara:** Conceptualization (equal); data curation (equal); investigation (equal); methodology (equal); project administration (equal); resources (equal); supervision (lead); validation (lead); writing – review and editing (equal). **Christopher McCarthy:** Conceptualization (supporting); investigation (supporting); methodology (supporting); resources (supporting); validation (equal); writing – review and editing (equal). **Innocent Pangapanga‐Phiri:** Conceptualization (supporting); investigation (supporting); methodology (supporting); resources (supporting); validation (equal); writing – review and editing (equal).

## CONFLICT OF INTEREST STATEMENT

The authors declare no conflict of interest.

## Data Availability

All occurrence data used in this study are provided in the paper.
